# Diagnosis of Alzheimer’s disease‐ Analysis of cross‐species biomarkers

**DOI:** 10.1002/alz.095406

**Published:** 2025-01-09

**Authors:** Shveta Bathla

**Affiliations:** ^1^ Yale School of Medicine, New haven, CT USA

## Abstract

**Background:**

Alzheimer disease (AD) is a heterogeneous multifactorial neurodegenerative disorder pathologically characterized by Aβ plaques, NFT, neuroinflammation, and synaptic and neuronal loss. The nature of most sAD cases strongly argues for an environmental link. The prominent pathological manifestations in AD patients are abnormal levels of Aβ 1‐42 (Aβ42), total tau (t‐tau) and p‐tau in brain and biofluids. Here, our goal is to find pathologically conserved phosphorylated tau signatures that are common to humans, non‐human primates and rodents, which may be utilized as biomarker candidates and/or therapy targets.

**Method:**

In order to replicate the behaviors of AD in DKI mice (DKIhTau‐APPNL‐G‐F/NL‐G‐F), we employed a single‐step, antibody‐free method to extract tau from the prefrontal cortex, which was followed by quantitative phospho‐proteomics. We have further investigated p‐Tau changes in aged rhesus macaques which naturally develop amyloid and tau pathology at advanced ages. In the current study we have also tested whether chronic treatment with a GCPII inhibitor might also reduce tau hyperphosphorylation in aged rhesus monkeys with naturally‐occurring GCPII and tau pathology. Using an automated Lumipulse platform, we have further confirmed p‐tau (pT181 & pT217) changes in CSF (140) and plasma (∼200) in AD patients.

**Result:**

A total of 40 and 39 phosphorylation sites were measured in human and mouse tau respectively. In DKI mice, significant increase were seen in pT181, pS202, pS214, pT217, and pT396, among other markers. The western blot analysis of entorhinal cortex (ERC) of monkeys revealed higher expression level of pS214 (*P* = 0.0012), pT181 (*P* = 0.0004) and pT217 (*P* = 0.0235) in aged monkeys as compared to young ones which captured p‐tau signatures at very earlier stage of AD. Moreover, pT217Tau levels in dlPFC and ERC were considerably reduced in elderly macaques treated with the GCPII inhibitor, 2‐MPPA. In AD patients compared to cognitively normal, the CSF p‐tau181 concentration is considerably greater ROC‐0.9299). However, compared to plasms p‐tau181(ROC‐0.7556), plasma p‐tau217(ROC‐0.8997) demonstrated noticeably greater diagnostic accuracy.

**Conclusion:**

With the help of this study, which comprised two animal models as well as CSF and plasma from healthy and AD patients, we have developed a composite biomarker panel that shows great promise for early AD detection.

